# Music-integrated strength–proprioceptive training improves lower-limb performance and postural balance in adolescents with visual impairment: a randomized controlled trial

**DOI:** 10.1038/s41598-026-53232-w

**Published:** 2026-05-22

**Authors:** Rym Baccouch, Hana Maatoug, Rihab Borji, Achraf Ammar, Thouraya Fendri, Haithem Rebai, Sonia Sahli

**Affiliations:** 1 Research Laboratory ‘Sports Performance Optimization, LR09SEP01, National Center of Medicine and Sport Sciences (CNMSS), Tunis, Tunisia; 2 High Institute of Sport and Physical Education of Sfax, University of Sfax, Sfax, Tunisia; 3 Department of Training and Movement Science, Institute of Sport Science, Johannes Gutenberg-University Mainz, Mainz, Germany; 4 Research Laboratory, Molecular Bases of Human Pathology, LR19ES13, Faculty of Medicine of Sfax, University of Sfax, 3000 Sfax, Tunisia; 5 Department of Nutrition and Food Technology, School of Agriculture, The University of Jordan, Amman, Jordan; 6 Université d’Orléans, Université Paris-Saclay, CIAMS UR 4532, 45100 Orléans, France; 7 Université d’Orléans, SAPRèM, 45100 Orléans, France

**Keywords:** Visual impairment, Adolescents, Physical training, Music listening, Physical fitness, Health care, Neuroscience, Psychology, Psychology

## Abstract

Adolescents with visual impairment (VI) often exhibit reduced physical fitness (PF) and functional autonomy due to limited sensory input and restricted opportunities for physical activity (PA). While integrating music into exercise programs has been shown to enhance motor engagement and sensory compensation in other populations, its potential role within physical training for adolescents with VI remains underexplored. Therefore, this study investigated whether integrating music listening into a combined strength–proprioceptive physical training program could further enhance PF in adolescents with congenital VI. Fifty-nine volunteer participants were randomly assigned to a physical training group (PTG), music-physical training group (MPTG) and control group (CG). The sit-to-stand test (STST), the Killy test, the functional reach test (FRT), and the single-leg stance test (SLST) were conducted before and after 8 weeks of training to assess PF. Our results showed significant pre–post improvements in all assessed PF outcomes in both PTG and MPTG (all *p* < 0.001) following the intervention. While both PTG and MPTG demonstrated significant pre–post improvements, post-intervention scores in STST (36.80 ± 6.17 vs. 29.47 ± 3.23 reps), Killy test (77.42 ± 20.10 vs. 63.57 ± 21.16 s), and SLST (25.09 ± 4.33 vs. 20.31 ± 5.99 s) were significantly (*p* < 0.001; *p* < 0.01; *p* < 0.001 respectively) higher in the MPTG compared to the PTG. However, no significant between-group difference was observed for the FRT (42.04 ± 4.81 vs. 40.84 ± 5.82 cm) following the intervention. No meaningful changes were observed in the control group. These findings suggest that integrating music into strength–proprioceptive training may provide an additional benefit for selected functional capacities in adolescents with VI, which may in turn support greater autonomy in daily activities.

**Supplementary Information:**

The online version contains supplementary material available at 10.1038/s41598-026-53232-w.

## Introduction

Individuals with visual impairment (VI) have lower physical fitness (PF) level compared to their sighted counterparts^1,2^. For instance, equilibrium and lower trunk flexibility deficiency^3,4^, atypical muscle tension^5^ and lower isokinetic strength of knee muscles^6^ have been reported in individuals with VI compared to normally sighted ones. In adolescents with VI, measures of muscular power remain below expected levels and do not demonstrate the typical improvements associated with increasing age^7,8^. The diminished PF levels observed in children and adolescents with VI were mostly attributed to their atypical motor development^9^. In fact, a 3-year longitudinal study examined locomotor skills in youth with VI, revealing delayed and stagnated development and enduring motor skill limitations^8^. Moreover, it has been reported that over 80% of children with VI failed to reach age-appropriate PF standards^10^. This delayed motor development reported in adolescents with VI was attributed to the absence of sensory input through the visual pathway during infancy^9^ and to the limited opportunities provided to this disabled group to engage in physical activity (PA) and sports^11,12^. Indeed, it has been confirmed that youth with VI tend to have a more sedentary lifestyle compared to their sighted peers^13^. In light of international recommendations that youth aged 5–17 should engage in at least 60 min of moderate-to-vigorous PA daily^14^; a population-based analysis of 561 youth with VI, aged between 10 and 17 years old, found that only 18.7% met the recommended levels of daily PA, indicating that the majority fail to achieve these international guidelines^15^. Unfortunately, this sedentary behavior reported in adolescents with VI can result in further decrease in health-related PF components^16,17^. Given that PF is crucial to perform daily life activities efficiently^18^, the low PF level reported in individuals with VI can result in significant functional limitations^19^. These limitations may contribute to decreased autonomy, increased fall-related injury occurrence, and higher risks of morbidity and mortality, especially in individuals with congenital VI^20^.

Therefore, an extensive body of literature focused on examining the impact of various types of physical training on PF in individuals with VI^21–25^ In fact, it has been indicated that such interventions can improve key components of PF, including muscular strength, balance, coordination, and overall functional capacity^26–29^. To date, most studies have focused on single-modality interventions, targeting either strength development or sensorimotor function in isolation. While these approaches produce measurable benefits, they may not fully address the multifactorial nature of motor impairments associated with VI, which often involve concurrent deficits in neuromuscular control, proprioception, and postural regulation^24,25,30^. Emerging findings from other clinical and athletic populations suggested that combined training programs integrating strength and proprioceptive components may produce more comprehensive improvements in PF than single-component interventions^31^. Such combined approaches benefit from the synergy between muscular and sensorimotor adaptations^32^, which may be particularly relevant given the complex motor challenges experienced by youth with VI. Despite these promising indications, the application of combined strength–proprioceptive training in adolescents with VI remains largely unexplored, representing a critical gap in the literature and an opportunity for innovation in adapted PA programs.

Beyond combining various types of physical exercises, to optimize the benefits of PA, previous studies reported the interest of integrating various forms of sensory stimulation alongside conventional physical training, rehabilitation programs and adapted PA^33–38^. Importantly, individuals with VI rely more on non-visual sensory inputs, such as auditory, tactile and proprioceptive cues, to compensate for visual deficits to perform various motor tasks^39,40^ and to interact with their environment^33^. Recently, a qualitative study of a dance program showed that adolescents with VI learn movement through sound and touch, highlighting the role of alternative sensory inputs in supporting social interaction, enjoyment and PA engagement^24^. Considering these previous insights, exploring sensory-enriched training approaches in adolescents with VI, particularly within structured physical training interventions, may be effective at improving PF outcomes. Among sensory enrichment strategies, music is considered a key approach, defined as a structured auditory stimulus^35–37^. Indeed, music has been incorporated into exercise training programs across various populations; healthy individuals^41^ and patients with disabilities^35–37^ to enhance motor and cognitive functions, as well as psychological well-being and physiological health. However, evidence on the potential added value of integrating background music listening within structured physical training for adolescents with VI remains limited and inconclusive. For instance, it is plausible that integrating music listening into physical training may amplify sensory engagement and optimize motor outcomes in adolescents with VI. From a theoretical perspective, the potential benefits of music in adolescents with VI could be understood through mechanisms related to auditory–motor coupling and sensory substitution^42–44^. Auditory–motor coupling may enable auditory stimuli to facilitate global motor performance through activation of sensorimotor networks^42,43,45^, while sensory substitution processes may allow auditory input to partially compensate for the lack of visual feedback^44^. Therefore, examining whether augmenting a strength–proprioceptive training program with background music listening could enhance its benefits on PF components is of practical and theoretical interest. Consequently, the present study aimed to investigate the effects of incorporating music listening into a combined strength–proprioceptive training program on PF components in adolescents with VI. We hypothesized that integrating music listening would lead to greater improvements in selected PF parameters compared to the same training conducted without music.

## Methods

### Participants

To estimate sample size, G*Power was used with the statistical option “ANOVA: repeated measures, within-between interaction” to assess the group × session effect in the 3-group × 2-session design across the study outcomes. As there is a lack of published data concerning the combined effect of PA and music listening on physical performance in individuals with VI, a medium effect size (Cohen f = 0.25), was assumed^46^. The parameters entered in the G*Power were: α = 0.05, power = 0.80, number of groups = 3, number of measurements = 2 sessions correlation among repeated measures = 0.50, and non-sphericity correction (ε) = 1. Based on these values, G*Power estimated that a total sample size of 42 participants would be sufficient to minimize the risk of a Type II error.

**Fig. 1:**
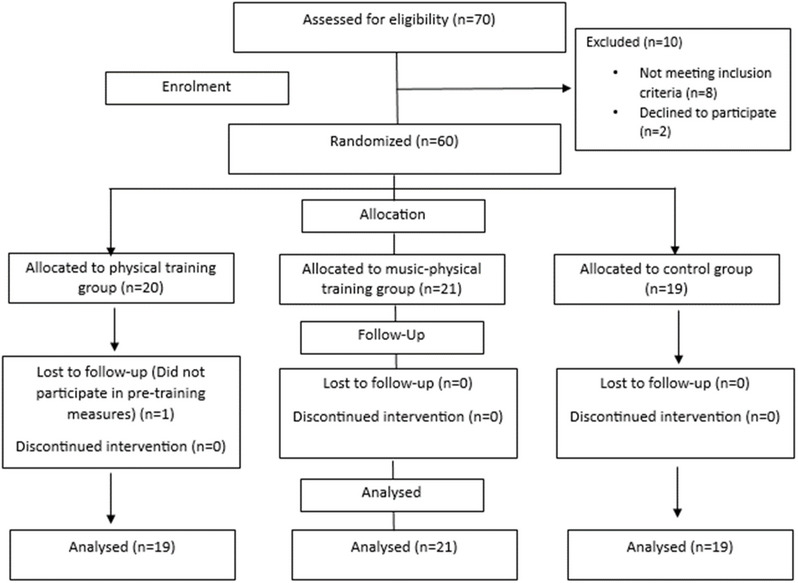
The flowchart of participants’ enrolment.

Our recruitment strategy involved a three-step screening process. Initially, 70 students were enrolled from the local high school of individuals with VI who were able to practice PA without any exception. In the subsequent stage, 60 of these screened students met our inclusion criteria and were chosen for this study (Fig. 1). To ensure that all participants met al.l inclusion criteria, the medical files were checked, the validated version of the international physical activity questionnaire (IPAQ) was filled by the examiner, the weight and body mass index (BMI) of all participants were measured using an electronic impedance meter (Tanita BC-545 N, Tokyo, Japan) and the height was measured with a tape measure. The included participants were adolescents with normal body weight (5th percentile < BMI< 85th percentile)^47^. All participants had a congenital severe VI, in both eyes, with visual acuity between 6/60 and 3/60 in the better eye^48^. Participants had low weekly activity level (IPAQ score < 600 MET)^49,50^. They participated in a 2-hour physical education session once a week but did not engage in any physical training/or music therapy programs on a regular basis. Participants with neurological diseases, musculoskeletal alterations, vestibular disorders, hearing impairment and orthopedic surgeries within the past 12 months were excluded. In the final stage, one student was excluded because he did not participate in pre-training measures. Consequently, 59 students were included in the final analyses (Fig. 1). Using a simple randomization method based on a randomization table created by a computer software program (Excel), an independent researcher randomly assigned participants to one of the three groups: physical training group (PTG) (*n* = 19, 10 males and 9 females), music-physical training group (MPTG) (*n* = 21, 12 males and 9 females) and the control group (CG) (*n* = 19, 11 males and 8 females) (Fig. 1). This study’s time frame is from January 2023 to March 2023.

### Study design

This randomized controlled trial was conducted in accordance with the Declaration of Helsinki. The study protocol was prospectively reviewed and approved by the local ethics committee, the Committee for the Protection of Persons of the South, Sfax, Tunisia, on 25 January 2022 under approval number CPP SUD No. 0382/2022. The study was conducted in accordance with the Consolidated Standards of Reporting Trials (CONSORT) guidelines and retrospectively registered on Pan African clinical trials registry (ID: PACTR202402852117450; URL: https://pactr.samrc.ac.za/; date of registration: 20/02/2024). Participant recruitment and all experimental procedures were initiated only after ethics approval had been obtained. However, registration occurred after completion of data collection due to administrative and logistical delays associated with conducting the study within a school setting and the time required to complete documentation for registry submission. Importantly, the study protocol, eligibility criteria, intervention procedures, outcome measures and statistical analysis plan were established prior to participant recruitment and remained unchanged throughout the study. No additional outcomes were introduced, no prespecified outcomes were omitted, and no deviations from the initial analysis plan occurred. All experiments were performed in accordance with relevant guidelines and regulations. Informed consent was obtained from the guardians of all participants prior to their inclusion in the study, and all data were anonymized to ensure confidentiality. All measurement procedures were carried out in the local high school of students with VI. All participants followed a familiarization session 48 h before any measurement to ensure a clear understanding of the test instructions. The intervention assignment followed a parallel design. In fact, the PTG underwent 8 weeks of strength-proprioceptive training, while the MPTG underwent the same physical training program combined with music listening during the same period. The CG took part in no intervention and continued their normal daily activities. To evaluate the PF level, the sit-to-stand test (STST), killy test, functional reach test (FRT), and single-leg stance test (SLST) were conducted in two test sessions: the first conducted 48 h before the intervention (pre-intervention) and the second 48 h after completing the eight-week intervention (post-intervention). All measurements were conducted by the same two experimenters, both PhD-level specialists in adapted PA. They worked together during each session and jointly administered all measurement procedures. During testing, one experimenter provided instructions and supervised task execution, while the other recorded and scored performances in real time. Although inter-rater reliability could not be assessed due to single-examiner scoring, a standardized protocol was strictly followed, and identical instructions were delivered across all participants and conditions to ensure consistency. All tests were conducted in a quiet, well-lit indoor room with stable lighting and minimal external distractions. The floor was flat, hard, and non-slippery, ensuring safe and uniform testing conditions for all participants. This study was conducted as a single-blind trial. Experimenters were blinded to group allocation, whereas participants and the trainer were not blinded, as it was not possible to mask the music-based training intervention.

### Intervention program

The training program was implemented twice weekly in the morning on non-consecutive days in a gymnasium, with each session lasting 45–60 min. All training sessions were conducted by the same adapted PA trainer, who provided verbal instructions and ensured correct execution of the exercises to confirm adherence to the protocol. The trainer was supported by the students’ regular adapted physical education teacher, an experienced and qualified instructor with over eight years of experience in the high school for students with VI, who assisted in ensuring safety and supervising all training settings.

Each session consisted of a warm-up phase (10 min), followed by the main program phase including various exercises (Table 1) and a cool-down phase (5 min)^51^. Each session was composed of six exercises targeting strength and proprioceptive components (Table 1). In order to stimulate proprioceptive afferents, selected exercises (Exercises 1–6, 8 and 9) were performed on both stable (firm surface) and unstable (foam surface) conditions. In addition, a proprioceptive cushion was used during the standing on one foot exercise (Exercise 7). A periodized training program^52^ was followed throughout the training period: three sets of 15 repetitions in the 1st and 2nd weeks, three sets of 20 repetitions in the 3rd and 4th weeks, four sets of 20 repetitions in the 5th and 6th weeks, and five sets of 20 repetitions in the 7th and 8th weeks^53–55^ with rest intervals of 90 s between sets^56,57^. The training intensity was monitored via the Borg scale^58^. Both training programs were performed at moderate-to-vigorous intensity (Borg CR-10 3–5), with mean session rating of perceived exertion (RPE) of 4.27 ± 0.16 for PTG and 4.09 ± 0.06 for MPTG. Each session was concluded with a recovery phase comprising stretching exercises. The MPTG performed the same training program as the PTG but with the addition of listening music during the training sessions. For the main training program, musical tracks with a fast tempo ranging from 120 to 140 beats per minute (BPM) were selected, as this range has been shown to enhance arousal, motivation and exercise adherence^59^. These psychophysiological responses have been associated with the ergogenic effects of music^60^, and may support motor performance during exercise^61,62^. In the present context, they may have contributed to the greater improvements observed in some PF outcomes among adolescents with VI. In addition, an average music tempo of 100 BPM was maintained during the warm-up and cool-down phases to create a supportive environment for muscle preparation and recovery^63^. The music playlist consisted of familiar tracks selected based on participants’ preferences. Specifically, participants were asked to provide a list of music they regularly listened to prior to the intervention, and all participants reported a preference for pop music. To reduce variability while preserving familiarity and motivation, the tempo of all selected tracks was verified using an online BPM analysis tool and only tracks within the predefined tempo ranges (120 to 140 BPM for the main training program; 100 BPM for the warm-up and cool-down) were included (see supplementary material file). A single standardized playlist was subsequently created for the MPTG using the “Audacity” software (version 3.7.6), including stimulative and energetic tracks. Each session included a continuous playlist corresponding to the duration of the training session. Music was delivered through portable JBL (James Bullough Lansing) Flip 5 speakers at a standardized, comfortable listening volume and served as background auditory stimulation. Participants were not instructed to synchronize their movements with the musical rhythm. All sessions were conducted in the morning in the same gymnasium. For both groups, participants were deemed to have successfully completed the training program if they attended at least 90% of the sessions.

**Table 1:** Summary of the training program exercises.

Exercise	Description
1. Air squat	The participant was asked to lower the hips from a standing position and then stand back up.
2. Squat jumps	From a standing position, the participant was asked to perform a knee flexion and extension up
3. Two-foot ankle hop	The participant was asked to stand straight up and hop repeatedly in place using only the ankle joint
4. Single-foot side-to-side ankle hop	The participant was asked to stand on one leg then started hopping back and forth between the initial position
5. Standing long jump	The participant began the exercise with standing with feet slightly apart and then jumped, as far as possible, landing on both feet without falling backwards
6. Standing on one foot	The participant was asked to stand on one foot on a firm surface
7. Standing on one foot on a proprioceptive cushion	The participant was asked to stand on one foot on a proprioceptive cushion
8. Lateral jump with both foot	The participant was asked to stand on two legs with their hands on the waist. Then he was asked to hop to the side, with maintaining the balance, and hop back to the starting position
9. Lateral jump with one feet	Same above mentioned description but with one leg
10. Straight sit ups	From a lying flat position with the legs straight and the hands placed behind the head, the participant was asked to pull his upper body to a sitting position, then slowly lower his upper body back down to the ground
11. Power sit-ups	The same above mentioned instructions but with bent knees
12. Flutter kicks	Starting from a supine position with arms by their sides, participants were instructed to extend their legs and perform small, rapid, up-and-down scissor-like motions

## Measurements

### Sit to stand test

In order to assess the lower limb functional strength, the one-minute STST was used^64^. It is a simple and reliable functional test that has been validated in children and adolescents with good test–retest reliability^65^ and that is widely used among both healthy children and those with chronic conditions^66^. During the test, participants were instructed to stand up and to sit down as many times as possible within 1 min, using a chair without armrests and measuring 46 cm in height^67^. Participants were asked to keep their feet parallel and to put their hands crossed over the chest in order to ensure that they would not use their arms to assist the movement. In addition, they were instructed to straighten their knees and stand up completely, and to touch the chair with the buttocks and to keep the angle of their knee joints approximately to 90° when sitting down^65^. The instructor told the participants to complete as many STS repetitions as possible within 1 min without any encouragement. After familiarization with the test, only one trial was conducted, as suggested by a systematic review of procedures^68^. The number of fully completed STS cycles for 1 min was recorded for analysis. Standardized instructions were provided to all participants across testing sessions.

### Killy test

To assess the endurance of participants’ lower limbs, the killy test was used in this study^69^. Participants were requested to sit with their back against the wall, arms at the sides, head touching the wall, knees flexed at 90°, with the hips also flexed at 90°. The test consists of maintaining this position as long as possible and stops when the participant takes off his head from the wall, gets up or collapses. Prior to the test, the examiner verified the correct alignment of the knees and hips and marked the position of the right heel on the floor. The participant then rested for approximately 30 s to minimize any fatigue from assuming the position. Afterwards, they were asked to reposition themselves using the floor mark as a reference. The knee angle was quickly rechecked before starting the measurement^70^. The time (s) during which the participant maintained the position was recorded using a stopwatch. The criterion for stopping the test was the loss of thigh horizontality. A first warning about the position is allowed. After familiarization with the test, only one trial was conducted. The recorded performance corresponded to the time taken to hold this position. Standardized instructions were provided to all participants across all testing sessions.

### Functional reach test

This test has been used in children with poor vision to evaluate dynamic postural balance^71^. During the FRT, participants were asked to point their arms in front of them in a straight line, and the reach value was recorded. Then, they were asked to lean forward without lifting their heels. The maximum distance (cm) to which the participants could lean forward without losing their balance or taking a step, was recorded. For each participant, three trials were conducted, and the average value was used for analysis^72^. Brief rest periods of approximately 5 to 10 s were allowed between trials^73^. Standardized instructions were provided to all participants across testing sessions.

### Single leg stance test

The SLST is a static balance test that was used previously among children with poor vision^71^. During the test, participants were asked to stand on one leg while maintaining an upright posture. In this position, the participant lifted the non-supporting leg off the ground without touching the other. The test was terminated if the lifted leg touched the ground or the other leg, or if the participant skipped, hopped, or grabbed surrounding objects to maintain balance. The test was effectuated on the dominant limb, defined as the preferred limb used to kick a ball. The duration (s) of the test was recorded using a stopwatch^71^. Three trials were conducted for each participant. The best performance of the three trials was used in analysis with rest period of 1–5 min between trials^74^. Standardized instructions were provided to all participants across testing sessions.

### Statistical analysis

Statistical analyses were performed using STATISTICA 12 software. The values are expressed as mean ± standard deviation (SD). The normality of the data distributions and the homogeneity of variance were verified using the Shapiro–Wilk test and Levene’s test, respectively. A one-way Analysis of variance (ANOVA) was used to compare the age, height, weight, BMI and IPAQ scores between the three groups of participants. The PF (STST, killy test, FRT and SLST) performances were analyzed using two-way ANOVAs [group as a between-subjects factor (3 levels: PTG/MPTG/CG) × session as a within-subjects factor (2 levels: pre-/post-intervention)].When significant differences were observed, a post-hoc analysis was then performed with the Bonferroni test in order to compare the experimental data two by two. The level of significance for all statistical analyses was set at *p* < 0.05. The effect size of each outcome measure was calculated using partial eta squared (η_p_^2^) interpreted as small (0.01–<0.06), medium (0.06–<0.14), or large (≥ 0.14)^75^. In addition, the 95% confidence interval (CI) were calculated^76^.

## Results

### Demographic characteristics of participants

There were no significant differences between the 3 groups in terms of age, height, weight, BMI and IPAQ scores (Table 2).

**Table 2:** Participants characteristics (mean ± standard deviation) of the physical training group *(PTG)*, the music-physical training group *(MPTG)* and the control group *(CG)*.

Characteristics	PTG	MPTG	CG	*P* value
Age (years)	16.68 ± 1.73	16.61 ± 1.93	16.21 ± 2.07	0.71 NS
Height (cm)	167.68 ± 11.29	165.52 ± 9.00	166.78 ± 10.99	0.8 NS
Weight (kg)	56.32 ± 12.11	55.25 ± 9.10	55.68 ± 10.85	0.95 NS
Body mass index (BMI) (kg/m^**2**^**)**	20.08 ± 4.20	20.19 ± 3.13	20.18 ± 4.27	0.96 NS
IPAQ scores (MET.min/week)	535.71 ± 143.01	550.38 ± 126.06	559.86 ± 163.57	0.85 NS

IPAQ: international physical activity questionnaire, NS: no significant difference (*p* > 0.05).

### Physical fitness performances

#### Sit to stand test

The two-way ANOVA with repeated measures revealed a significant main effect of the group (F _(2,36)_ = 36.16, *p* < 0.001, η_p_^2^ = 0.66) and session (F _(1,18)_ = 421.22, *p* < 0.001, η_p_^2^ = 0.95) factors, as well as a significant group x session (F _(2,36)_ = 405.35, *p* < 0.001, η_p_^2^ = 0.95) interaction on the STST scores. The post hoc results showed that there was no significant difference (*p* > 0.05; CG vs. PTG; 95% CI: [3.78, 7.41], CG vs. MPTG; 95% CI: [6.16, 11.64], PTG vs. MPTG; 95% CI: [6.61, 12.49]), between the three groups, before training (Table 3). After training, the STST scores increased significantly in both PTG (*p* < 0.001; 95% CI: [3.22,6.31]) and MPTG (*p* < 0.001; 95% CI: [2.72, 5.15]) (Table 3). These scores were significantly greater in the MPTG compared to the PTG after the training period (*p* < 0.001; 95% CI: [7.64, 14.43]). The STST scores remained unchanged in the CG after the training period (*p* > 0.05; 95% CI: [2.39, 4.67]) (Table 3).

**Table 3:** Physical fitness measures (mean ± standard deviation) in the physical training group (*PTG*), the music-physical training group (*MPTG*) and the control group (*CG*) during the pre-intervention and the post-intervention sessions.

	Pre-intervention			Post-intervention		
	PTG	MPTG	CG	PTG	MPTG	CG
STST score (reps)	15.36 ± 4.62	15.95 ± 4.94	15.05 ± 4.12	29.47 ± 3.23***	36.80 ± 6.17***###	15.36 ± 4.62
Killy test (s)	25.31 ± 4.87	24.28 ± 3.88	24.21 ± 5.42	63.57 ± 21.16***	77.42 ± 20.10***##	24.36 ± 4.25
FRT (cm)	30.57 ± 3.15	29.80 ± 3.55	30.26 ± 4.09	40.84 ± 5.82***	42.04 ± 4.81***	30.00 ± 3.26
SLST (s)	9.47 ± 2.26	10.47 ± 2.52	9.05 ± 2.41	20.31 ± 5.99***	25.09 ± 4.33***###	10.15 ± 1.57

STST: Sit to stand test, FRT: Functional reach test, SLST: Single leg stance test, min: minute, reps: repetitions, s: seconds, cm: centimeter. ***. Significant difference compared to pre-intervention session (*p* < 0.001), ##. Significant difference compared to the PTG (*p* < 0.01), ###. Significant difference compared to the PTG (*p* < 0.001).

### Killy test

The two-way ANOVA with repeated measures showed a significant group (F _(2,36)_ = 39.66, *p* < 0.001, η_p_^2^ = 0.68) and session (F _(1,18)_ = 219.42, *p* < 0.001, η_p_^2^ = 0.92) main effects and group x session (F _(2,36)_ = 63.68, *p* < 0.001, η_p_^2^ = 0.77) interaction on the killy test time. Before training, the post hoc test showed that there were no significant differences between the three groups in the killy test performance (*p* > 0.05; CG vs. PTG; 95% CI: [5.55, 10.86], CG vs. MPTG; 95% CI: [6.93, 13.08], PTG vs. MPTG; 95% CI: [6.92, 13.06]) (Table 3). After training, the killy test time increased significantly in both PTG (*p* < 0.001; 95% CI: [13.46, 26.34]) and MPTG (*p* < 0.001; 95% CI: [14.44, 27.27]) with higher time in the MPTG compared to the PTG (*p* < 0.01; 95% CI: [24.11, 45.51]) (Table 3). The killy test time remained unchanged in the CG after the training period (*p* > 0.05; 95% CI: [2.35, 4.60]) (Table 3).

### Functional reach test

The two-way ANOVA with repeated measures revealed a significant effect of the group (F _(2,36)_ = 12.15, *p* < 0.001, η_p_^2^ = 0.4) and session (F _(1,18)_ = 258.3, *p* < 0.001, η_p_^2^ = 0.93) factors, and a significant group x session (F _(2,36)_ = 74.25, *p* < 0.001, η_p_^2^ = 0.8) interaction on the FRT performance. Before training, the baseline distances were similar for the three groups (*p* > 0.05; CG vs. PTG; 95% CI: [3.89, 7.62], CG vs. MPTG; 95% CI: [7.76, 14.65], PTG vs. MPTG; 95% CI: [7.66, 14.46]). After training, the Bonferroni test showed a significant increase in the FRT distance, compared to pre-training, for both PTG (*p* < 0.001; 95% CI: [2.69, 5.28]) and MPTG (*p* < 0.001; 95% CI: [2.95, 5.57]), with no difference between the MPTG and the PTG (*p* > 0.05; 95% CI: [10.68, 20.15]). However, for the CG, the FRT distances did not change (*p* > 0.05; 95% CI: [2.02, 3.96]) after the training period (Table 3).

### Single leg stance test

The two-way ANOVA with repeated measures demonstrated a significant main effect of the group (F _(2,36)_ = 36.63, *p* < 0.001, η_p_^2^ = 0.67) and session (F _(1,18)_ = 277.01, *p* < 0.001, η_p_^2^ = 0.93) factors, and a significant group x session (F _(2,36)_ = 60.24, *p* < 0.001, η_p_^2^ = 0.76) interaction on SLST time. The post Hoc test showed that, before the training, there were no significant (*p* > 0.05; CG vs. PTG; 95% CI: [3.02, 5.92], CG vs. MPTG; 95% CI: [3.37, 6.37], PTG vs. MPTG; 95% CI: [3.30, 6.24]) differences between the three groups (Table 3). After the training, the SLST time increased significantly in the PTG (*p* < 0.001; 95% CI: [3.62, 7.09]) and MPTG (*p* < 0.001; 95% CI: [3.35, 6.33]) with significantly (*p* < 0.001; 95% CI: [7.25, 13.07]) higher times in the MPTG compared to the PTG (Table 3). The SLST time remained unchanged (*p* > 0.05; 95% CI: [1.15, 2.25]) in the CG after the training period (Table 3).

## Discussion

The current study aimed to investigate the effects of an 8-week physical training program in combination with music listening, compared to physical training program without music, on PF in adolescents with VI. Results from this study demonstrated that both training approaches enhanced PF level in adolescents with VI for all measured components. Notably, the combination of music with physical training produced additional benefits compared to physical training without music in improving muscle strength, muscle endurance, and static balance ability.

Overall, given the improvements in both training groups compared to the CG, it can be suggested that physical training, combining strength and proprioception, is an effective intervention for adolescents with VI for PF level enhancement. These findings align with previous research on the impact of strength-proprioceptive training on various physical performances across various populations^54,77–79^. Moreover, it has been reported that strength training on unstable surfaces significantly improves PF in adolescents, young adults, and older adults without VI^80^. In adolescents with VI, the beneficial effect of physical training on motor outcomes has been proven extensively^25,27–29^. However, the effect of a combined strength-proprioceptive training on PF hasn’t been investigated previously. Concerning muscle functional strength and endurance of the lower limbs, our findings showed a significant improvement in both trained groups, while the CG exhibited no changes in these muscular capacities. Our findings are in accordance with previous research, that demonstrated lower limb muscle strength improvement following a combined strength-proprioceptive training in individuals with intellectual disabilities^54^, children with Autism Spectrum Disorders^31^ and middle-aged women^78^. While our study did not examine the underlying mechanisms behind these changes, several explanations have been suggested in earlier studies. In fact, strength exercises have been suggested to induce particular nervous adaptations including faster recruitment and more coordinated activation of motor units, increased neuronal firing rates, and enhanced intramuscular and intermuscular coordination^81,82^. As individuals with VI frequently experience muscle weakness^6^, which is attributed to disruptions in neural systems^83^ and slower sensory and motor interactions^1^, engaging in strength exercises may therefore contribute to enhancing muscular performance, by targeting these underlying deficits.

Furthermore, in the current study, the improvements in dynamic (FRT) and static (SLST) postural balance observed in both trained groups, compared to the untrained group, could be attributed to the beneficial effects of proprioceptive exercises integrated into the combined strength-proprioceptive training. In fact, throughout proprioceptive exercises, somatosensory inputs could be modified by changing the support surface, requiring participants to recalibrate the remaining inputs efficiently within the central nervous system^84,85^. Indeed, standing on a foam surface is thought to challenge somatosensory information^86,87^ by decreasing the reliability of plantar cutaneous mechanoreceptive information^88^. In addition, it has been demonstrated that proprioceptive training enhances the body’s movement awareness by increasing signals from foot and muscle receptors during swaying motions, especially during muscles co-contraction that may effectively improve postural balance abilities^89^. Given that muscular effectiveness, particularly in lower extremities, is crucial for balance control^90^, improvements in lower limb muscular strength following the training period may contribute to enhanced balance abilities, as confirmed by the results of the STST after the training period in both trained groups. In fact, in normally sighted individuals, similar results were found and stipulated that combined balance-strength training improves balance and muscle power in elderly people^91^.

The major result of our study demonstrated that the MPTG had superior improvements compared to the PTG, specifically showing higher gains in lower limb functional strength, muscle endurance, and static postural balance. Thus, these results suggested an additional beneficial effect of music on PF level in adolescents with VI. In previous literature, similar studies investigating the effect of adding music listening to physical training were conducted in both healthy^92^ and individuals suffering from disabilities^37,93^. However, the current study was the first to investigate this effect in individuals with VI. Similarly to our findings, a facilitator effect of exercise-music training on PF was found in patients with Alzheimer’s disease^37^. In addition, a rehabilitation program based on gymnastic training combined with music has been shown to effectively improve locomotor skills, flexibility, coordination, and balance in children with Autism Spectrum Disorder and Down syndrome^93^. Moreover, in healthy children, balance training combined with music improved balance and muscle strength^92^. The greater enhancement of PF observed in the MPTG, compared to the PTG, may be explained by several potential mechanisms. Auditory–motor coupling mechanism could underlie the observed benefits in the group trained with music^42,43,45^. In fact, it has been reported that background music could engage functional connectivity between auditory and motor systems, even in the absence of visible movement, reflecting intrinsic auditory–motor coupling^42^. It may therefore be suggested that, even when movement is not synchronized with musical beats, beneficial effects may still arise through these underlying auditory–motor coupling mechanisms. Through this mechanism, listening to music, as an external provided auditory stimulus, may enhance movement organization by facilitating interactions between auditory sensory input and brain motor planning regions^94^. These interactions have been proposed to enhance motor neuron firing and muscle fiber recruitment, potentially leading to stronger muscle contractions^95^. These effects could partially account for the greater improvement of the lower limb functional strength observed in the MPTG compared to the PTG. In addition, sensory substitution mechanism may contribute to these effects. In the absence of reliable visual feedback, auditory cues can partially compensate by providing continuous information about timing and movement dynamics, thus supporting postural regulation and movement planning^44^. Indeed, individuals with VI rely more heavily on auditory information due to reduced visual input; this compensatory strategy may promote cross-modal plasticity, leading to stronger integration between auditory and motor systems^43,96^. This compensatory reliance on auditory input may further enhance the effectiveness of externally provided stimuli, such as the background music implemented in the present physical training program for adolescents with VI. In previous research examining music-related PA among adolescents with VI, a 3-week capoeira dance program reported that participants learned movements through rhythmic music, verbal guidance, and tactile methods such as hand-under-hand instruction^33^. These sensory-enriched experiences, together with supportive interactions with instructors and volunteers, enhanced engagement, enjoyment, a sense of freedom, and confidence in PA^33^. Indeed, the capoeira-based intervention used rhythmic music, whereas the present study used background music. Background music may still facilitate sensory–motor connections even without clear rhythmic cues^42^; however, it may be less effective at enhancing movement synchronization and coordination^97,98^.

Overall, listening to music has been associated with the activation of brain areas such as the left inferior frontal gyrus and the insular cortex, which are involved in attention and perception^99,100^. Such neural activity may support cognitive processing and motor organization^101^. As it has been established that postural balance control requires attentional resources and cognitive processing^102,103^, incorporating music into training routines may therefore facilitate attentional engagement, contributing to improvements in static postural balance. Considering the importance of muscular output to maintain postural balance^90^, the higher performances observed in the MPTG compared to the PTG when executing the SLST may also be linked to greater strength gains following the combined music-exercise intervention, as reflected by STST results. Music has been considered a potentially effective strategy for modulating physical performance through both physiological and psychological mechanisms^62^. It is plausible that music may influence physiological arousal, including heart rate regulation and catecholamine release^104^, which could contribute to enhanced physical performance. Moreover, music might promote motivation and emotional well-being through increased dopamine secretion, potentially facilitating greater engagement in PA^105,106^. In addition, listening to music may help reduce perceived fatigue and improve mood, allowing individuals to sustain effort for longer durations^59,107^. These physiological and psychological responses may partly explain the greater improvements observed in the MPTG compared to the PTG, particularly in endurance tasks such as the Killy test. In the current study, music was selected primarily based on a stimulating fast tempo to enhance energy and motivation^59^ as well as participants’ familiarity with the tracks. Familiarity may influence emotional responses to music, as recognizable songs can enhance mood through positive associations^37,108^. These psychophysiological effects may collectively contribute to the ergogenic potential of music^60^ and may partly explain the greater improvements in PF observed in adolescents with VI who participated in the music-integrated training program.

While MPTG showed greater improvements than PTG in lower-limb functional performance and static postural balance (SLST), no additional benefit of music was observed for dynamic balance, as assessed by the FRT. This result may be explained by the task-specific nature of the FRT, which primarily assesses limits of stability through a brief forward displacement of the center of mass rather than continuous postural regulation. Unlike tasks requiring sustained balance control, the FRT involves a short, discrete movement that does not rely heavily on rhythmic coordination or prolonged attentional engagement, potentially limiting the influence of music-related mechanisms such as increased arousal or sensorimotor entrainment^62^. Moreover, beyond lower limb strength, performance in this test depends largely on trunk flexibility, and anthropometric characteristics, which may be less responsive to auditory stimulation^109^. The physical training alone elicited substantial improvements in both groups, potentially limiting the observable additional effect of music. Moreover, the FRT may lack sensitivity to detect subtle enhancements in dynamic balance in adolescents with VI, as postural adaptations in this population depend on the integration of multiple sensory inputs and fine-tuning of postural strategies rather than maximal forward reach^3^. Therefore, the absence of an additive effect of music should be interpreted with caution and may reflect task specificity rather than the inefficacy of music-integrated training.

Although the present study suggests benefits of the training programs, it is important to note that the control group did not participate in a structured active intervention and instead continued their usual daily routines. As a result, some of the improvements observed in the PTG and MPTG may be partly explained by the higher levels of PA, supervision, and engagement, rather than solely by the specific effects of strength–proprioceptive or music-integrated training.

### Limitations

This randomized controlled trial offers several strengths, including a supervised, periodized strength–proprioceptive program and the comparison of two intervention arms with a usual-activity control group, allowing a first controlled investigation of music-integrated training in adolescents with congenital severe VI. The use of simple, functionally relevant fitness tests (STST, Killy test, FRT, SLST) also enhances practical applicability in school and rehabilitation contexts.

However, the findings should be interpreted with caution due to several limitations. First, although the sample size was adequate for detecting group-by-time differences in the selected outcomes, it remains relatively modest and was drawn from a single specialized high school, which may limit the generalizability of the results to other age ranges, VI severities, educational contexts, or cultural settings. Future studies with larger and more diverse samples are therefore needed to confirm and extend these findings. Second, only PF outcomes were assessed; physiological, psychological and cognitive variables (e.g., heart rate, motivation, enjoyment, perceived effort, attentional focus,.) were not included, which limits understanding of the underlying mechanisms through which music may have contributed to performance improvements. Future studies should integrate validated physiological, psychometric or neurocognitive assessments to explore whether the improvements were due to physiological mechanisms, cognitive engagement, emotional responses or motivational states. In addition, the assessment of PF was primarily limited to lower limb strength and balance measures; although these components are fundamental for daily functional performance in adolescents with VI, other relevant domains such as aerobic capacity, coordination, gait parameters, and functional mobility were not evaluated. Including these outcomes in future studies would provide a more comprehensive understanding of the effects of music-integrated training on overall functional fitness. Third, the intervention lasted eight weeks without any post-intervention follow-up; thus, it remains unclear whether these improvements are sustained over the medium or long term. Conducting follow-up assessments (e.g., 3–6 months later) would help determine the durability and real-life applicability of the observed effects. Long-term longitudinal designs would also allow examination of whether these adaptations translate into sustained functional and behavioral changes. Fourth, given the nature of the music intervention, blinding of participants and the trainer was not feasible, that may potentially introduce performance and expectation biases. These factors should be considered when interpreting the results and planning future studies. Fifth, the control group continued their normal daily activities and did not participate in any structured physical training intervention, while this design leads to observe training effects, it may overestimate the intervention effect. In addition, no music-only condition was included, therefore, the specific contribution of music, beyond its combination with exercise, cannot be fully isolated. Future studies should consider including an active control group performing an alternative PA (e.g., balance-only or strength-only exercises) and a placebo-type intervention (e.g., music-only) to strengthen the internal validity of the findings. Sixth, although fast-tempo popular music was used and tempo was standardized, music was not individualized based on motivational qualities. In addition, because the playlist was derived from participant-preferred familiar music, some residual variability in musical characteristics across tracks may have remained despite tempo standardization and the use of a single standardized playlist. Future studies should compare tightly standardized playlists with individually optimized music selections to determine whether familiarity, motivational salience, or specific musical features differentially influence training responses. Future research should also investigate the effects of different music genres and tempos to better understand how specific musical characteristics may influence intervention effectiveness. Finally, although background music used in the present study can positively influence selected PF components, it may not optimally facilitate auditory–motor entrainment compared with rhythmic music. Implementing rhythmic music in physical training programs could provide additional benefits by enhancing motor coordination through the synchronization of movement to rhythmic cues. Future programs may also consider other sensory cues stimulation, such as tactile guidance and partner interactions, to examine their combined effects on motor-related PF components in adolescents with VI.

### Practical implications

The current findings support the feasibility of implementing music-integrated strength–proprioceptive training in real-world settings, particularly in schools for students with VI, rehabilitation centers, and adapted physical education programs. Indeed, this intervention requires minimal and accessible equipment (e.g., audio devices and basic exercise materials), making it cost-effective and easy to execute. Moreover, training sessions can be conducted in familiar and controlled environments, which is essential for adolescents with VI. Notably, instructors do not require highly specialized musical expertise; however, they should be trained in adapted PA principles and in the appropriate use of auditory cues to guide movement and ensure safety. The integration of music may be adapted in terms of tempo, rhythm, and volume to match participants’ abilities and training objectives. Overall, this approach represents a simple, accessible, and attractive strategy that can be readily incorporated into PA programs for adolescents with VI.

## Conclusion

In conclusion, combined strength–proprioceptive training appears to be a promising approach for improving PF in individuals with VI, as both intervention groups showed meaningful gains relative to the control condition. The addition of music was associated with greater improvements in lower-limb functional strength, muscular endurance, and static balance, although no additional benefit was observed for dynamic balance as assessed by the FRT. From a practical perspective, music may represent a simple, accessible, and low-cost adjunct to adapted physical education and school-based training programs, with potential to enhance selected functional capacities and support autonomy in daily life among adolescents with VI.

## Supplementary Information

Below is the link to the electronic supplementary material.

Supplementary Material 1

## Data Availability

Data associated with this paper can be produced on request from the first author.
