# Supplementary material for: Music-integrated strength–proprioceptive training improves lower-limb performance and postural balance in adolescents with visual impairment: a randomized controlled trial
Source: Sci Rep. 2026 May 22;16:15984. doi: 10.1038/s41598-026-53232-w (PMC13197423; doi:10.1038/s41598-026-53232-w)
Supplement: Supplementary file 1 — Supplementary Material 1 [file 41598_2026_53232_MOESM1_ESM.docx]

| Title | Artist | Style/Genre | Tempo | Rhythm |
| --- | --- | --- | --- | --- |
| Dima Labess | Klay BBG | Tunisian Rap/Pop | 130 BPM | 4/4, repetitive hip-hop groove |
| Sugar | Maroon 5 | Pop/Funk-pop | 120 BPM | 4/4, funky upbeat groove |
| C’est la vie | Khaled | Raï/Pop | 128 BPM | 4/4, raï-dance groove |
| Bochret Khir | Hussain Al Jassmi | Arabic Pop | 130 BPM | 4/4, energetic march-like groove |
| Waka Waka | Shakira | Pop/Latin-Pop | 127 BPM | 4/4, energetic syncopated groove |
| Tukoh Taka | Myriam Fares | Arabic Pop | 135 BPM | 4/4, club-style dance groove |
| Gangnam Style | Psy | K-Pop | 132 BPM | 4/4, electronic dance groove |
| Don’t Start Now | Dua Lipa | Disco-Pop | 124 BPM | 4/4, disco groove |
| Bad Habits | Ed Sheeran | Pop | 126 BPM | 4/4, electronic dance groove |
| Counting Stars | OneRepublic | Pop | 122 BPM | 4/4, syncopated percussive groove |
| It Ain’t Me | Kygo & Selena Gomez | Pop | 100 BPM | 4/4, tropical house groove |
| Galbi | Samara | Urban Pop | 100 BPM | 4/4, urban groove |
| Can’t Feel My Face | The Weeknd | Pop/Funk-Pop | 100 BPM | 4/4, funk-pop groove |

List of music selected by participants from their own playlists
